# Publication Performance and Trends in Dental Anxiety Research: A Comprehensive Bibliometric Analysis

**DOI:** 10.1155/ijod/5530537

**Published:** 2026-01-19

**Authors:** Yuh-Shan Ho, Robert Vautard, Robert Schibbye, Nikolaos Christidis

**Affiliations:** ^1^ CT HO Trend, 3F.-7, No. 1, Fuxing N. Rd., Songshan Dist., Taipei City, Taiwan; ^2^ Laboratoire des Sciences du Climat et de l’Environnement, Institut Pierre-Simon Laplace, Université Paris-Saclay, Gif sur Yvette, France, universite-paris-saclay.fr; ^3^ Division of Pediatric Dentistry, Department of Dental Medicine, Karolinska Institutet, Huddinge, Sweden, ki.se; ^4^ Division of Oral Rehabilitation, Department of Dental Medicine, Karolinska Institutet, Huddinge, Sweden, ki.se

**Keywords:** anxiety disorders, bibliometrics, dental anxiety, dental phobia, dentistry

## Abstract

**Objective:**

This bibliometric analysis aimed to systematically evaluate publication performance and identify evolving trends in dental anxiety research over the past three decades, providing a structured overview of key research topics and thematic progression.

**Methods:**

The study analyzed 1556 articles indexed in the Science Citation Index Expanded database from 1991 to 2024. Data extraction included titles, abstracts, author keywords, and *Keywords Plus*. The analytical approach incorporated bibliometric indicators such as total citations, citations per publication, and annual publication trends. A word analysis technique identified five major research topics and their evolution across three distinct periods: 1991–2011, 2012–2019, and 2020–2024.

**Results:**

Analysis highlighted significant growth in dental anxiety research publications, particularly in recent years, reflecting increased global interest. The study identified five main thematic areas: etiology and risk factors, clinical presentation and consequences, prevalence and assessment tools, treatment and preventive interventions, and pediatric dentistry. The trends indicated a growing emphasis on multidisciplinary approaches, like cognitive–behavioral therapy (CBT) and adjunctive therapies such as virtual reality and aromatherapy. Pediatric dentistry consistently emerged as a critical field, underscoring the importance of early interventions.

**Conclusion:**

This bibliometric review demonstrated substantial advancements in understanding dental anxiety, emphasizing multidisciplinary treatments and tailored pediatric interventions. Future research should focus on integrating novel therapeutic strategies and refining preventive measures to mitigate dental anxiety effectively.

## 1. Introduction

Dental anxiety is a prevalent yet often underrecognized phenomenon that affects individuals across the lifespan, exerting significant influence on both oral health outcomes and general well‐being [1, 2]. Despite its widespread impact, the term dental anxiety is variably defined and encompasses a broad spectrum of emotional and behavioral responses. Dental phobia, however, is a formally recognized medical diagnosis with established diagnostic criteria characterized by marked and persistent fear related to dental settings that significantly impair functioning. In this manuscript, the term dental anxiety is consistently used as an overarching construct to describe the research field as a whole, unless otherwise specified. The term dental fear refers to milder anticipatory responses described in population‐based surveys, whereas dental phobia is reserved exclusively for the clinically diagnosable condition with established diagnostic criteria.

Despite modern advances in dental care, dental anxiety associated with dental treatment persists as significant barriers to regular dental visits, often leading to avoidance behaviors, compromised oral health, and a diminished quality of life [3, 4]. Recognizing the complexity and variability of dental anxiety is essential for delivering appropriate, individualized treatment, preventing the development of anxiety, and ultimately improving patient health outcomes. A bibliometric analysis is particularly useful in this context because dental anxiety lies at the intersection of psychological and clinical domains, resulting in a highly heterogeneous and rapidly expanding literature. Traditional narrative reviews struggle to capture the breadth, evolution, and interdisciplinarity of this research field [5]. A systematic mapping of publication trends, influential contributions, and thematic shifts, therefore, provides unique insight into how psychological mechanisms and clinical management strategies have codeveloped overtime. Notably, despite the longstanding relevance of dental anxiety, no previous comprehensive bibliometric analysis has been dedicated exclusively to this topic, highlighting a gap this study aims to address.

Dental anxiety manifests differently across the lifespan. Among children, dental anxiety is associated with uncertainty and lack of familiarity with dental procedures, environments, or staff. Young children typically fear the unknown, but this fear frequently subsides with age, experience, and increased cognitive understanding of dental routines [6, 7]. Conversely, persistent dental anxiety in adults is linked to previous traumatic dental experiences, negative interpersonal interactions within dental or healthcare settings, or other unrelated psychological traumas [8, 9]. Such adults frequently demonstrate pronounced avoidance behaviors, often resulting in severe oral health deterioration [10]. Among older adults, cognitive impairments, sensory deficits, and broader health‐related anxiety can further complicate the management of dental anxiety. In this population, anxiety may be exacerbated by reduced cognitive resilience, sensory hypersensitivity, or misunderstandings about treatment procedures [11].

Effective management of dental anxiety requires age‐appropriate and individually tailored approaches. In pediatric populations, dental behavior strategies emphasize building trust and familiarity through management techniques such as tell‐show‐do, positive reinforcement, and parental involvement [6, 12]. Adolescents and adults with high dental anxiety and dental phobia benefit from psychological interventions, including exposure‐based cognitive–behavioral therapy (CBT), systematic desensitization, relaxation techniques, and pharmacological aids such as sedation [3, 13]. Older patients, particularly those with cognitive decline, may require additional supportive measures, clear communication strategies, and possibly anxiolytic medications under careful supervision to ensure compliance and comfort during treatment [11]. An understanding of dental anxiety throughout the lifespan, coupled with the implementation of evidence‐based treatment and preventive modalities, is essential for improving oral health outcomes and ensuring patient comfort and compliance.

Bibliometric studies have shown that structured analyses of research trends can guide the development of clinical interventions and prioritize resources effectively, aligning with evolving psychological insights and patient care requirements [5, 14]. The continuous expansion of scientific literature has significantly advanced our understanding of dental anxiety; however, the sheer volume of available publications creates substantial challenges for clinicians and researchers striving to stay updated with the most relevant and influential findings [3, 13]. Navigating this extensive literature can feel like searching for a needle in a haystack, as vital research can easily become obscured [15].

To manage this complexity, bibliometric analyses have emerged as essential tools. By systematically evaluating publication data, bibliometrics provides comprehensive insights into the progression and thematic evolution of dental anxiety research [5, 16]. In the context of dental anxiety, bibliometric analysis offers valuable perspectives for developing evidence‐based clinical strategies and interventions [1]. This analytical method aids in identifying influential studies and emerging trends, guiding clinical practice, and informing research priorities and funding decisions [17, 18]. Utilizing bibliometric insights allows dental practitioners, researchers, and healthcare policymakers to effectively manage the growing body of scientific literature, focusing their efforts on evidence‐based practices and tailored interventions designed to mitigate dental anxiety and improve patient outcomes [19, 20].

In this context and unlike existing bibliometric studies by our group in related dental fields (i.e., temporomandibular disorders, bruxism, and dental education) the present analysis is the first to focus exclusively on dental anxiety. Thus, the present bibliometric study aims to address the existing challenges by analyzing research trends and publication performance specifically in the field of dental anxiety. This provides a unique and field‐specific mapping of thematic evolution over three decades. The purpose of this bibliometric analysis is not only to conduct a comprehensive citation performance evaluation but also to employ innovative bibliometric methods. Specifically, the analysis incorporates detailed evaluations of article titles, author keywords, *Keywords Plus* [21], and abstracts [22], providing a richer and more nuanced understanding of research trajectories and influential contributions in dental anxiety.

## 2. Materials and Methods

The data utilized in this study were obtained from the Web of Science Core Collection (WoSCC) provided by Clarivate Analytics, specifically from the online version of the Science Citation Index Expanded (SCI‐EXPANDED). The dataset was last updated on May 1, 2025. Boolean operators such as OR and AND were employed when formulating the search queries. To ensure accurate retrieval, multiword terms were enclosed in quotation marks (“ ”). The OR operator was used to identify records that contained at least one of the specified keywords within the Topic field, which encompasses the title, abstract, author keywords, and *Keywords Plus*.

The search was conducted using targeted keywords, including “dental anxiety,” “dental fear,” and “dental phobia.” To improve retrieval sensitivity and ensure comprehensive coverage of the literature, a broad range of less frequently used or alternative terms was also included [23]: “fear of dental,” “anxiety in dental,” “anxiety during dental,” “dental fears,” “fear dental,” “dental fear anxiety,” “dental trait anxiety,” “fearful dental,” “dentally fearful,” “odontophobia,” “anxiety among dental,” “anxiety about dental,” “dentophobia,” “fear of dentistry,” “fear of dentists,” “anxiety in dentistry,” “anxiety on dental,” “odontophobics,” “anxiety before dental,” “fear during dental,” “anxiety of dental,” “anxiety toward dental,” “anxiety towards dental,” “dental anxieties,” “dental care anxiety,” “dental care fear,” “dental patient anxiety,” “dental treatment fear,” “dentist fear,” “dentophobic,” “phobia and dental,” “anxiety regarding dental,” “dental anxiety phobia,” “dental patients’ anxiety,” “dental phobias,” “dentist phobia,” “dentists’ anxiety,” “dentists’ fear,” “fear about dental,” “fear for dental,” “fear of dentist,” “fearful of dental,” “fears about dental,” “fears in dental,” “odontophobic,” “phobia of dental,” “anxiety from dental,” “anxiety with dental,” “dental state anxiety,” “dental treatment phobia,” “dentophobics,” “feared by dental,” “anxieties about dental,” “anxiety about dentistry,” “anxiety among dentistry,” “anxiety around dental,” “anxiety following dental,” “anxiety on dentists,” “anxiety towards dentists,” “anxiety‐eliciting dental,” “dental and anxiety,” “dental fearful,” “dental hygiene fears,” “dental injection phobia,” “dental procedure anxiety,” “dental state fear,” “dental trait fear,” “dental treatment anxieties,” “dental needle phobia,” “dental‐related anxieties,” “dental‐related fear,” “dental‐specific anxiety,” “dentist anxiety,” “dentist for fear,” “dentists allay fear,” “dentists for fear,” “dentists through phobia,” “fear among dentists,” “fear in dentistry,” “fearful about dentistry,” “fearful of dentists,” “fearing dental,” “fears of dental,” “phobia in dentistry,” “phobias about dentistry,” “phobias within dentistry,” “fear of intraoral injections,” “intraoral injection fear,” “intraoral injection phobia,” “specific phobia dentist,” and “specific phobia dentistry” in SCI‐EXPANDED were also considered. Together, this approach ensured both precision and breadth in capturing dental anxiety‐related publications.

A total of 2073 documents, including 2066 documents (99.7% of 2073 documents), containing search keywords in the terms of TOPIC, were searched‐out in the SCI‐EXPANDED database published between 1991 and 2024.

The complete records of documents retrieved from SCI‐EXPANDED, including annual citation counts, were downloaded into Microsoft 365 Excel for analysis. Following the methodology previously described by our research group [24, 25], manual coding was performed to refine and enrich the dataset. Additionally, journal impact factors (IF2023) were obtained from the 2024 edition of the Journal Citation Reports (JCRs).

In 2011, the use of the “front page,” comprising the title, abstract, and author keywords, was introduced as a filtering strategy to improve search precision when using the Topic (TS) field in the WoSCC for bibliometric research [26, 27]. This filtering approach improves precision by focusing on key textual elements, thereby minimizing irrelevant retrieval. When applied to medical research topics within SCI‐EXPANDED, the front‐page filter revealed significant discrepancies, highlighting its effectiveness in enhancing data quality. For example, its implementation resulted in a 4.0% reduction in irrelevant records in dental education [28], a 15% deviation in studies on temporomandibular disorders [15], and a notable 31% deviation in bruxism research [29]. The search for documents containing the search keywords in their “front page” yielded a total of 1884 documents, which accounted for 91% of the 2066 initially identified documents.

In the WoSCC database, the reprint author is identified as the corresponding author; however, in this study, the term “corresponding author” was used instead [30]. For articles with a single author, single institution, and single country without explicitly defined authorship roles, the sole author was designated as both the first and corresponding author, and the institution and country were classified accordingly [24]. In instances where multiple corresponding authors were indicated, all listed corresponding authors, along with their respective institutions and countries, were included in the analysis [24]. Furthermore, for articles in SCI‐EXPANDED that provided only addresses without naming specific affiliations, these addresses were verified and updated to reflect the appropriate institutional affiliations [24].

Following the methodology described by Chiu and Ho in 2005 [31], affiliations from England, Scotland, Northern Ireland, and Wales were consolidated under the designation of the United Kingdom (UK). Similarly, affiliations listed as Turkiye and New Caledonia were reclassified as Turkey [32] and France [33], respectively. Affiliations from Hong Kong were categorized under China [27]. Additionally, affiliations originally recorded as Yugoslavia were reviewed and reclassified as either Slovenia or Serbia based on institutional details [34].

The evaluation of publications in this study was conducted using three citation indicators:

• *C*_year_: This indicator represents the number of citations received from the WoSCC in a specific year (i.e., *C*_2024_ denotes the citation count for the year 2024) as proposed by Ho in 2012 [19].
• TC_year_: This indicator reflects the total number of citations received from the WoSCC from the year of publication until the end of the most recent year (2024 in this study; denoted as TC_2024_), as introduced by Wang et al. in 2011 [35].
• CPP_year_: The average number of citations per publication, calculated as CPP_2024_ = TC_2024_/TP, where TP denotes the total number of publications. This measure was suggested by Ho in 2013 [36].

The citation indicators can be applied for a wide range of categories, for example, total and annual publications, as well as publications in a document type, language, Web of Science category, journal, country, institution, author, and an article.

In 2014, six publication indicators were proposed to evaluate the publication performance of countries and institutions [37, 38] as:

1. TP: Total number of articles published.
2. IP: Number of articles published by a single country (IP_C_) or institution (IP_I_).
3. CP: Number of internationally collaborative articles (CP_C_) or interinstitutionally collaborative articles (CP_I_).
4. FP: Number of first‐author articles.
5. RP: Number of corresponding‐author articles.
6. SP: Number of single‐author articles.

Moreover, six citation indicators (CPP_2024_) corresponding to these publication indicators were used to evaluate the impact of publications on document types, journals, countries, and institutions, as proposed by Ho and Mukul in 2021 [39].

### 2.1. Statements

#### 2.1.1. Statement About Originality

The research conducted in this manuscript is original, not presently under consideration for publication elsewhere, free of conflict of interest and conducted by the highest principles of human subject welfare. The authors alone are responsible for the content and writing of the paper.

#### 2.1.2. Ethics Statement

Not applicable since data was publicly available.

#### 2.1.3. Patient Consent

Not applicable since no patient data is reported.

## 3. Results and Discussion

Throughout Section 3, the term dental anxiety is used as an umbrella term reflecting the scope of the bibliometric analysis, unless distinctions between dental fear and dental phobia are explicitly stated.

### 3.1. Characteristics of Document Types and Languages

To evaluate the characteristics of document types within a specific research field, key bibliometric indicators such as the average number of citations per publication per year (CPP_year_) and the average number of authors per publication (APP) are commonly employed [40]. Between 1991 and 2024, a total of 1884 documents related to dental anxiety were indexed in the SCI‐EXPANDED database, encompassing 11 different document types, as summarized in Table 1. Among these, research articles were the most prevalent, accounting for 1556 documents (83% of the total), with an average of 4.4 APP. Of all document types, reviews (122 publications) exhibited the highest citation impact, with a CPP_2024_ of 30, based on 3683 total citations. This made reviews, on average, 1.4 times more cited than research articles. This citation ratio between reviews and articles is slightly lower than that observed in other dental research domains. For example, in dental education, reviews were cited 1.8 times more frequently than articles [28], while in the fields of temporomandibular disorders and bruxism, the ratio was 1.7 [15, 29]. However, in third molar research, reviews were cited less frequently than articles, with a CPP_year_ that was only 0.73 times that of articles [41].

**Table 1:** Citations and authors according to the document type.

Document type	TP	%	TP ^∗^	AU	APP	TC_2024_	CPP_2024_
Article	1556	83	1556	6892	4.4	31,688	20
Meeting abstract	147	7.8	147	481	3.3	11	0.075
Review	122	6.5	122	616	5.0	3683	30
Editorial material	34	1.8	32	65	2.0	103	3.0
Proceedings paper	17	0.90	17	74	4.4	403	24
Early access	14	0.74	14	65	4.6	27	1.9
Letter	10	0.53	9	14	1.6	1	0.10
News item	9	0.48	6	6	1.0	1	0.11
Correction	5	0.27	5	20	4.0	7	1.4
Discussion	1	0.053	1	1	1.0	0	0
Retracted publication	1	0.053	1	10	10	22	22

*Note:* TP: total number of publications; TP ^∗^: total number of publications with author information in the SCI‐EXPANDED database; %: percentage of articles in all articles; AU: total number of authors; APP: average number of authors per publication; TC_2024_: total number of citations from WoSCC since publication year until the end of 2024; CPP_2024_: average number of citations per publication (TC_2024_/TP).

Abbreviation: N/A, not available.

Within the corpus of 1884 dental anxiety‐related publications, 45 were identified as highly cited papers, defined as having a total citation count (TC_2024_) of 100 or more [42]. This group comprised 37 articles and eight reviews.

It should be noted that documents in the WoSCC can be assigned to multiple document types. For example, 39 proceedings papers, 10 retracted publications, and one early access paper were all simultaneously classified as articles. Consequently, the cumulative percentages in Table 1 exceed 100% [43].

Different document types contribute to bibliometric analyses in distinct ways. Among them, research articles, that are typically structured with sections including introduction, methods, results, discussion, and conclusion, are generally regarded as the most significant for evaluating scholarly output in a given field [44]. In the case of dental anxiety research, a total of 1556 articles were published across four languages. English was overwhelmingly dominant, representing 1550 articles (99.6%), followed by German (four articles), and one article each in Croatian and Dutch.

### 3.2. Characteristics of Publication Outputs

**Figure 1:**
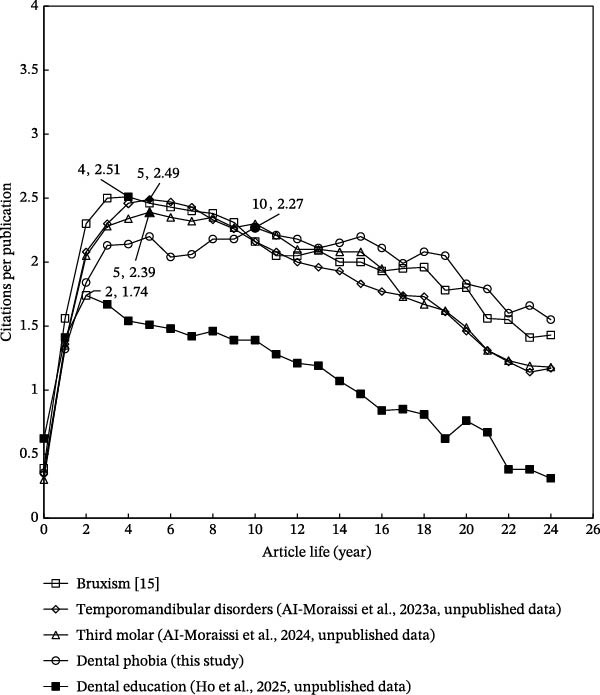
Citations per publication by article life. This trend suggests that dental anxiety research not only attracts early attention but maintains long‐term relevance, indicating the stability and consistency of scientific interest in the field.

To gain a deeper understanding of the citation lifespan of research articles, Chuang et al. [45] proposed analyzing the relationship between the average number of citations per article in a given year (as indexed in WoSCC) and the age of the article, defined as the number of years since publication. Figure 1 presents a comparative analysis across various dental‐related research topics. In the field of dental anxiety, 1556 articles received a total of 550 citations in their publication year (*C*_0_ = 550), yielding an average of 0.353 citations per publication (CPP_0_ = 0.353). Citation impact peaked in the tenth year after publication, during which 703 articles accumulated 1595 citations, resulting in the highest CPP_10_ of 2.27 (1595/703). This pattern suggests enduring interest in the field, although the peak was slightly lower than that observed in other dental research areas. For example, bruxism exhibited a CPP_4_ of 2.51 in the fourth full year postpublication [29], temporomandibular disorders reached a CPP_5_ of 2.49 [15], and third molar research had a CPP_5_ of 2.39 [41]. In contrast, dental education demonstrated a lower long‐term citation impact, with a CPP_2_ of only 1.74 [28], despite having the highest initial citation impact. Of the 3483 dental education articles, 2164 citations were received in the year of publication, resulting in the highest CPP_0_ across all topics at 0.621. This was followed by bruxism (0.389), dental anxiety (0.353), temporomandibular disorders (0.348), and third molar research (0.300). These findings indicate that while dental education articles attract early attention, their long‐term citation impact is comparatively modest. Conversely, articles on dental anxiety demonstrate a more extended citation lifespan, reflecting sustained academic interest.

**Figure 2:**
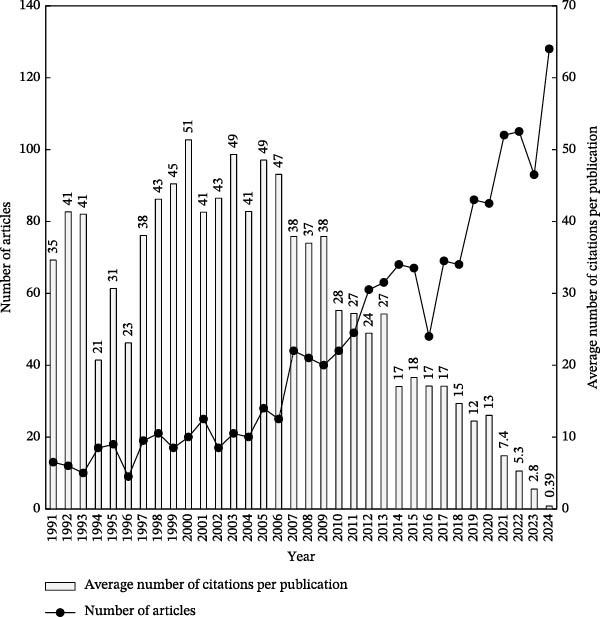
Number of dental anxiety‐related articles and average number of citations per publication by year. This pattern demonstrates a sustained increase in both research output and scholarly influence, reflecting the growing global attention to dental anxiety as a clinically and psychologically significant issue.

**Figure 3:**
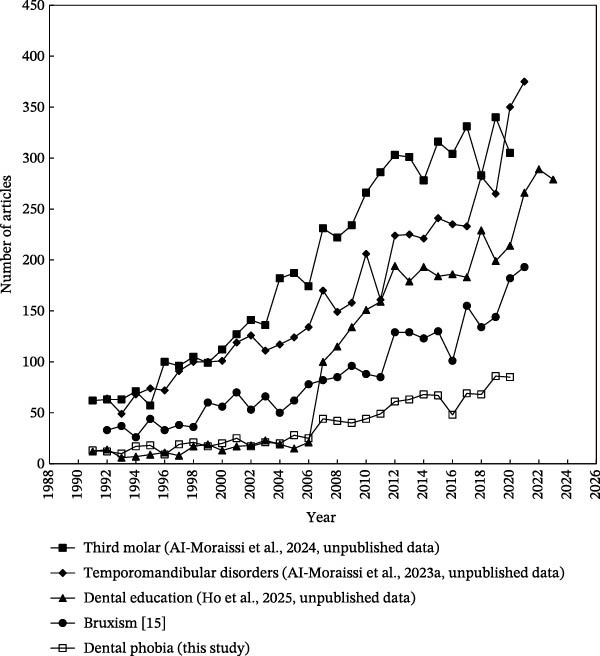
Compares the development trends for five dental‐related topics. The divergence in trajectories highlights how dental anxiety has become more multidisciplinary overtime, with certain themes gaining prominence as clinical and psychological perspectives increasingly intersect.

To assess cumulative citation impact from the year of publication through the end of 2024, the citation indicator CPP_2024_ was used. This metric allowed for an exploration of the relationship between CPP_2024_ and the annual number of publications (TP) by year, offering insights into both research impact and publication trends within the topic [36]. The annual number of dental anxiety‐related articles indexed in SCI‐EXPANDED was counted and is illustrated in Figure 1. It is widely recognized that citation accumulation requires time. For example, 128 articles published in 2024 had received a total of 50 citations by the end of the same year, resulting in a CPP_2024_ of 0.39 (50 citations/128 articles). In contrast, 20 articles published in 2000 accumulated 1027 citations over the same period, yielding a CPP_2024_ of 51 (1027 citations/20 articles). As shown in Figure 2, CPP_2024_ generally stabilizes approximately 16 years after publication. Articles related to dental anxiety show a more prolonged citation impact than those in other dental topics. For instance, studies in dental education [28] and temporomandibular disorders [15] typically reached their citation plateau within approximately 10 years, as shown in Figure 3.

A notable increase in the number of publications was observed, rising from 48 articles in 2016–128 in 2024. It is likely that this pattern reflects a growing global interest in the topic, with research activity expanding beyond the traditionally dominant countries such as Sweden, Norway, Australia, the Netherlands, the United Kingdom, and the USA. Over the past decade, particularly in the last 5 years, there has been a notable increase in publications from countries such as India, China, and those with Arabic and Persian‐speaking populations. While output from the “classical” countries that have historically demonstrated high comparative research output remained relatively stable, the sharp rise in contributions from these emerging regions represents an important and intriguing development.

### 3.3. Web of Science Category and Journal

In 2023, the JCRs indexed 9486 journals within the Science Citation Index Expanded (SCI‐EXPANDED), spanning 178 Web of Science categories. Recent studies have established baseline metrics to characterize these categories [46] and individual journals [47], using indicators such as the average number of citations per publication (CPP_year_) and the average number of APP. Within SCI‐EXPANDED, a total of 263 journals across 65 Web of Science categories published articles related to dental anxiety. The top 10 most productive categories are summarized in Table 2. Among these, the category dentistry, oral surgery, and medicine, which included 91 journals in 2023, accounted for the largest share, publishing 1147 articles, that is, approximately 74% of the total 1556 articles on dental anxiety. This substantial proportion reflects the concentrated disciplinary focus of research in this area.

**Table 2:** The top 10 most productive Web of Science categories.

Web of Science category	TP (%)	No. *J*	APP	CPP_2024_
Dentistry, oral surgery, and medicine	1147 (74)	91	4.2	22
Public, environmental, and occupational health	208 (13)	207	4.3	32
Pediatrics	196 (13)	130	4.4	16
General and internal medicine	95 (6.1)	167	4.7	9.1
Psychiatry	49 (3.1)	157	5.0	15
Neurosciences	34 (2.2)	270	4.5	23
Health care sciences and services	30 (1.9)	108	5.7	22
Research and experimental medicine	30 (1.9)	136	5.7	11
Multidisciplinary sciences	29 (1.9)	72	5.8	11
Environmental sciences	23 (1.5)	275	4.5	11

*Note:* TP: total number of publications; No. *J*: number of journals in a category in 2023; APP: average number of authors per publication; CPP_2024_: average number of citations per publication (TC_2024_/TP).

Among the top 10 categories listed in Table 2, the category public, environmental, and occupational health, comprising 207 journals, achieved the highest CPP_2024_, with an average of 32 citations per publication. In contrast, the category general and internal medicine, with 167 journals, had a CPP_2024_ of 9.1. In terms of authorship patterns, the category multidisciplinary sciences exhibited the highest APP, with an average of 5.8 APP across 29 articles. Meanwhile, despite its high output, the dentistry, oral surgery, and medicine category showed a slightly lower APP of 4.2 across 1147 articles. Overall, this distribution highlights the strong dominance of dentistry‐oriented journals while also underscoring the growing interdisciplinary interest in dental anxiety across public health, psychiatry, and general medicine.

Table 3 presents the 11 most productive journals in the field, along with their 2023 impact factor (IF_2023_), average number of citations per publication (CPP_2024_), and average number of APP. All 11 journals are indexed under the dentistry, oral surgery, and medicine category. The journal *Community Dentistry and Oral Epidemiology* (IF_2023_ = 1.8) published the highest number of articles, contributing 123 articles, which accounts for 7.9% of all 1556 dental anxiety‐related articles. This was followed by the journals *European Journal of Oral Sciences* and *BMC Oral Health*.

**Table 3:** The top 11 most productive journals.

Journal	TP (%)	IF_2023_	APP	CPP_2024_
Community Dentistry and Oral Epidemiology	123 (7.9)	1.8	3.9	43
European Journal of Oral Sciences	106 (6.8)	1.8	4.2	28
BMC Oral Health	101 (6.5)	2.6	5.0	14
Acta Odontologica Scandinavica	86 (5.5)	1.4	3.8	22
British Dental Journal	83 (5.3)	2.0	3.5	23
International Journal of Paediatric Dentistry	77 (4.9)	2.3	4.7	20
European Journal of Paediatric Dentistry	32 (2.1)	2.2	4.5	10
Journal of Clinical Pediatric Dentistry	32 (2.1)	1.5	4.6	10
Community Dental Health	29 (1.9)	0.90	3.3	12
Journal of Oral and Maxillofacial Surgery	29 (1.9)	2.3	4.3	18
Journal of the American Dental Association	29 (1.9)	3.1	4.1	47

*Note:* TP: total number of articles; %: percentage of articles in all articles; IF_2023_: journal impact factor in 2023; *R*: rank in Web of Science category of dentistry, oral, and surgery medicine; APP: average number of authors per publication; CPP_2024_: average number of per publication (TC_2024_/TP).

Among the top journals, *the Journal of the American Dental Association* recorded the highest CPP_2024_, with an average of 47 citations per article. In contrast, *the European Journal of Paediatric Dentistry* (IF_2023_ = 2.2) published 32 articles, with a comparatively lower CPP_2024_ of 10. APP values among the top 11 journals ranged from 5.0 authors per article in the journal *BMC Oral Health* to 3.3 in the journal *Community Dental Health*.

Outside the top 11 in terms of productivity, several journals with limited contributions exhibited notably high impact factors. The journal *Advanced Functional Materials* had the highest IF_2023_ of 19, based on a single article. This was followed by the journals *Psychotherapy and Psychosomatics* (two articles; IF_2023_ = 16) and *the International Journal of Surgery* (one article; IF_2023_ = 13). Together, these findings indicate that while a small number of specialized dental journals dominate publication volume, citation impact varies substantially across journals.

### 3.4. Publication Performances: Countries and Institutions

It is widely recognized that the first and corresponding authors typically contribute most substantially to a research article [48]. At the institutional level, the affiliation of the corresponding author is often regarded as indicative of the study’s origin or home institution [19]. Within the SCI‐EXPANDED database, six dental anxiety‐related articles (0.39% of the 1556 articles) lacked affiliation data. The remaining 1550 articles were authored by researchers affiliated with institutions in 79 countries. Of these, 1261 articles (81%) were classified as single‐country publications, originating from 60 countries, and had an average of 20 citations per publication (CPP_2024_). The other 289 articles (19%) were produced through international collaborations involving authors from 69 countries, and these had a slightly higher CPP_2024_ of 21. These findings suggest that international collaboration may contribute modestly to increased citation impact in dental anxiety research.

Six publication indicators, along with their corresponding citation indicators (CPP_2024_), were used to compare the top 10 most productive countries in dental anxiety research, following the framework proposed by Ho and Mukul in 2021 [39]. These countries include five from Europe, two from the Americas, two from Asia, and one from Oceania (Table 4). Egypt, with 26 articles (ranked 20^th^ overall), was the most productive country from Africa.

**Table 4:** Top 10 productive countries.

Country	TP	TP (*n* = 1550)		IP_C_ (*n* = 1261)		CP_C_ (*n* = 289)		FP (*n* = 1550)		RP (*n* = 1544)		SP (*n* = 67)	
		*R* (%)	CPP_2024_	*R* (%)	CPP_2024_	*R* (%)	CPP_2024_	*R* (%)	CPP_2024_	*R* (%)	CPP_2024_	*R* (%)	CPP_2024_
UK	204	1 (13)	22	1 (11)	25	2 (22)	17	1 (10)	24	1 (10)	24	2 (16)	22
USA	183	2 (12)	24	3 (9.2)	24	1 (23)	24	3 (8.8)	25	3 (8.6)	24	3 (13)	28
Sweden	155	3 (10)	26	2 (10)	26	7 (8.7)	23	2 (9.1)	26	2 (9.2)	26	9 (3.0)	42
Turkey	99	4 (6.4)	15	4 (7.4)	15	26 (2.1)	17	4 (6.3)	15	4 (6.3)	15	4 (10)	18
Brazil	91	5 (5.9)	15	5 (5.3)	14	8 (8.3)	16	5 (5.4)	15	5 (5.4)	15	N/A	N/A
Netherlands	91	5 (5.9)	32	7 (4.7)	36	5 (11)	25	7 (4.8)	34	7 (4.9)	34	5 (6.0)	25
China	82	7 (5.3)	15	6 (5.1)	11	12 (6.2)	30	6 (5.0)	15	6 (5.2)	13	9 (3.0)	19
Australia	80	8 (5.2)	23	12 (3.1)	27	3 (14)	20	12 (3.3)	24	12 (3.4)	23	1 (19)	38
Norway	74	9 (4.8)	21	8 (4.3)	20	10 (6.9)	23	8 (4.1)	20	8 (4.1)	20	6 (4.5)	33
Germany	72	10 (4.6)	25	9 (3.6)	21	6 (9.3)	33	9 (3.7)	22	9 (3.8)	22	N/A	N/A

*Note:* TP: number of total articles; TP *R* (%): total number of articles and the percentage of total articles; IP_C_ *R* (%): rank and percentage of single‐country articles in all single‐country articles; CP_C_ *R* (%): rank and percentage of internationally collaborative articles in all internationally collaborative articles; FP *R* (%): rank and the percentage of first‐author articles in all first‐author articles; RP *R* (%): rank and the percentage of corresponding‐author articles in all corresponding‐author articles; SP *R* (%): rank and the percentage of single‐author articles in all single‐author articles; CPP_2024_: average number of citations per publication (CPP_2024_ = TC_2024_/TP).

Abbreviation: N/A, not available.

The United Kingdom led in four of the six publication indicators: total publications (TP = 204; 13% of the 1550 articles), single‐country publications (IP_C_ = 139; 11% of the 1261 single‐country articles), first‐author publications (FP = 156; 10% of the 1550 first‐author articles), and corresponding‐author publications (RP = 162; 10% of the 1544 corresponding‐author articles). The United States ranked highest in internationally collaborative publications (CP_C_), contributing 67 articles, or 23% of the 289 collaborative articles. Australia led in single‐author publications (SP), with 13 articles, accounting for 19% of the 67 single‐author articles.

Among the top 10 most productive countries listed in Table 4, the Netherlands exhibited the highest citation impact across four key publication indicators. With 91 total publications (TP), 59 single‐country publications (IP_C_), 75 first‐author publications (FP), and 75 corresponding‐author publications (RP), the Netherlands achieved the highest CPP_2024_ values of 32, 36, 34, and 34 citations per publication, respectively. Germany led in internationally collaborative publications (CP_C_), with 27 such articles achieving an average of 33 citations per publication, the highest in this category. Sweden, despite contributing only two single‐author articles (SP), recorded the highest CPP_2024_ for that indicator, with 42 citations per publication. These patterns illustrate the continued dominance of a limited number of high‐income countries in dental anxiety research, alongside a gradual diversification of research output through increasing international collaboration.

Of the 1550 analyzed dental anxiety‐related articles, 562 (36%) were single‐institution publications, with a CPP_2024_ of 21 citations per article, while the remaining 988 articles (64%) resulted from institutional collaborations, with a slightly lower CPP_2024_ of 20. This suggests that institutional collaboration did not enhance and may have slightly reduced, citation impact in this research area.

Table 5 presents the top 10 most productive institutions and their publication profiles. Among these, institutions from the Netherlands, Norway, and Sweden were each represented twice, while Australia, Finland, the United Kingdom, and the United States were each represented once. Notably, three institutions, that is, the University of Gothenburg (U Gothenburg, Sweden), the University of Amsterdam (U Amsterdam, Netherlands), and the University of Oulu (Finland), produced single‐author articles.

**Table 5:** Top 10 most productive institutions.

Institution	TP	TP (*n* = 1550)		IP_I_ (*n* = 562)		CP_I_ (*n* = 988)		FP (*n* = 1550)		RP (*n* = 1533)		SP (*n* = 67)	
		*R* (%)	CPP_2024_	*R* (%)	CPP_2024_	*R* (%)	CPP_2024_	*R* (%)	CPP_2024_	*R* (%)	CPP_2024_	*R* (%)	CPP_2024_
U Gothenburg	80	1 (5.2)	24	1 (4.1)	27	1 (5.8)	23	1 (3.2)	27	1 (3.2)	26	N/A	N/A
U Amsterdam	50	2 (3.2)	34	52 (0.36)	67	2 (4.9)	33	10 (1.2)	40	10 (1.2)	40	N/A	N/A
U Adelaide	43	3 (2.8)	30	4 (2.1)	45	5 (3.1)	24	12 (1.2)	36	10 (1.2)	34	1 (15)	48
U Oulu	40	4 (2.6)	21	21 (0.71)	16	3 (3.6)	21	6 (1.4)	23	6 (1.4)	25	N/A	N/A
KCL	39	5 (2.5)	22	3 (2.5)	23	10 (2.5)	22	6 (1.4)	21	3 (1.6)	20	9 (1.5)	12
U Washington	36	6 (2.3)	34	9 (1.2)	55	7 (2.9)	29	3 (1.5)	40	12 (1.2)	46	2 (6.0)	46
PDHS	35	7 (2.3)	27	N/A	N/A	4 (3.5)	27	32 (0.39)	18	64 (0.26)	23	9 (1.5)	70
ACTA	34	8 (2.2)	35	6 (1.8)	37	11 (2.4)	34	2 (1.7)	40	2 (2.0)	38	9 (1.5)	29
U Bergen	33	9 (2.1)	26	4 (2.1)	40	15 (2.1)	18	5 (1.5)	31	6 (1.4)	31	9 (1.5)	19
U Oslo	32	10 (2.1)	21	8 (1.6)	26	13 (2.3)	19	10 (1.2)	18	8 (1.3)	18	4 (3.0)	40

*Note:* TP: total number of articles; TP *R* (%): total number of articles and percentage of total articles; IP_I_ *R* (%): rank and percentage of single‐institution articles in all single‐institution articles; CP_I_ *R* (%): rank and percentage of interinstitutionally collaborative articles in all interinstitutionally collaborative articles; FP *R* (%): rank and percentage of first‐author articles in all first‐author articles; RP *R* (%): rank and percentage of corresponding‐author articles in all corresponding‐author articles; CPP_2024_: average number of citations per publication (CPP_2024_ = TC_2024_/TP). U Gothenburg, University of Gothenburg, Sweden; U Amsterdam, University of Amsterdam, Netherlands; U Adelaide, University of Adelaide, Australia; U Oulu, University of Oulu, Finland; KCL, King’s College London, UK; U Washington, University of Washington, USA; PDHS, Public Dental Health Service, Sweden; ACTA, Academic Centre Dentistry Amsterdam (Academisch Centrum Tandheelkunde Amsterdam), Netherlands; U Bergen, University of Bergen, Norway; U Oslo, University of Oslo, Norway.

Abbreviation: N/A, not available.

The U Gothenburg led in five of the six publication indicators: total publications (TP = 80; 5.2% of the 1550 articles), single‐institution publications (IP_I_ = 23; 4.1% of the 562 single‐institution articles), interinstitutional collaborations (CP_I_ = 57; 5.8% of the 988 collaborative articles), first‐author publications (FP = 49; 3.2% of the 1550 first‐author articles), and corresponding‐author publications (RP = 49; 3.2% of the 1533 corresponding‐author articles). The University of Adelaide (U Adelaide) led in single‐author publications (SP), contributing 10 articles, or 15% of the 67 single‐author articles.

Among the top 10 institutions, the Academic Centre for Dentistry Amsterdam (ACTA) had the highest CPP_2024_ for both total publications (TP = 34; CPP_2024_ = 35) and interinstitutional collaborations (CP_I_ = 24; CPP_2024_ = 34). The University of Washington (U Washington) recorded the highest citation impact for first‐author and corresponding‐author publications, with CPP_2024_ values of 40 (FP = 24) and 46 (RP = 18), respectively. The U Amsterdam had the highest CPP_2024_ for collaborative publications (CP_I_ = 48; CPP_2024_ = 67), while the Public Dental Health Service in Sweden (PDHS) led in citation impact for single‐author publications, with one article cited 70 times.

These findings are consistent with previous evidence indicating that the United States and United Kingdom along with, to a lesser extent, Australia and the Netherlands are recognized leaders in psychology and psychiatry research [49], subjects that converge with dental anxiety. Notably, the Nordic countries, despite their relatively small populations, have a disproportionately high impact on dental anxiety research. This is largely attributable to certain specific impactful institutions and individuals who have built strong research traditions in the field. At the institutional level, the results suggest that a relatively small number of research centers contribute disproportionately to high‐impact dental anxiety research, reflecting the presence of established research traditions and expertise.

Alongside these traditionally dominant research centers, the field of dental anxiety has recently shown a notable geographic expansion. The increasing number of publications from regions such as India, China, and Arabic‐ and Persian‐speaking countries may reflect several converging factors, including large and growing populations, expanded dental education systems, and increased investment in academic research. In addition, heightened interest in complementary and non‐pharmacological approaches to anxiety management may have contributed to the observed research focus in these regions. Collectively, these developments suggest a gradual broadening of dental anxiety research beyond its historically concentrated geographic core.

### 3.5. Citation Histories of the 10 Most Frequently Cited Articles

Total citation counts in the WoSCC are continually updated. To minimize potential bias associated with the dynamic nature of citation data, the total number of citations from the year of publication through the most recently completed year (TC_year_) was used, as recommended by Wang et al. in 2011 [35], to ensure a more comprehensive and consistent dataset.

**Figure 4:**
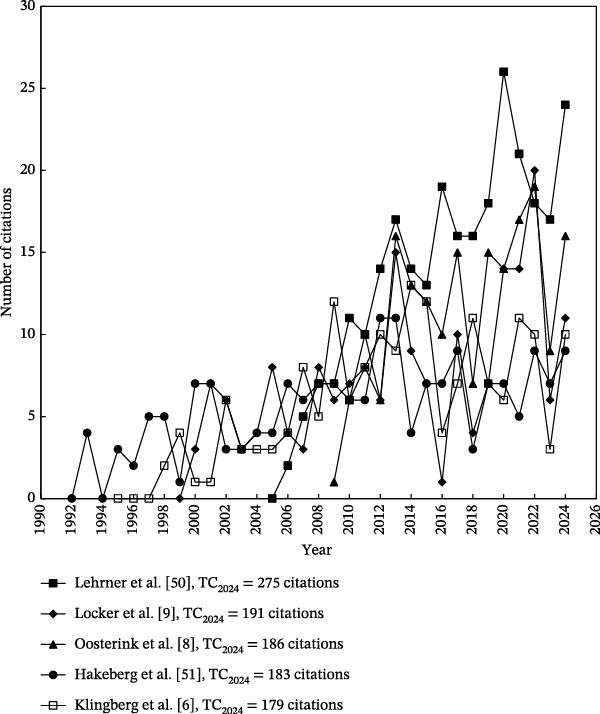
Citation histories of the top five most frequently cited articles. These citation trajectories indicate that foundational studies in dental anxiety continue to shape the field, underscoring their long‐term conceptual and methodological influence.

**Figure 5:**
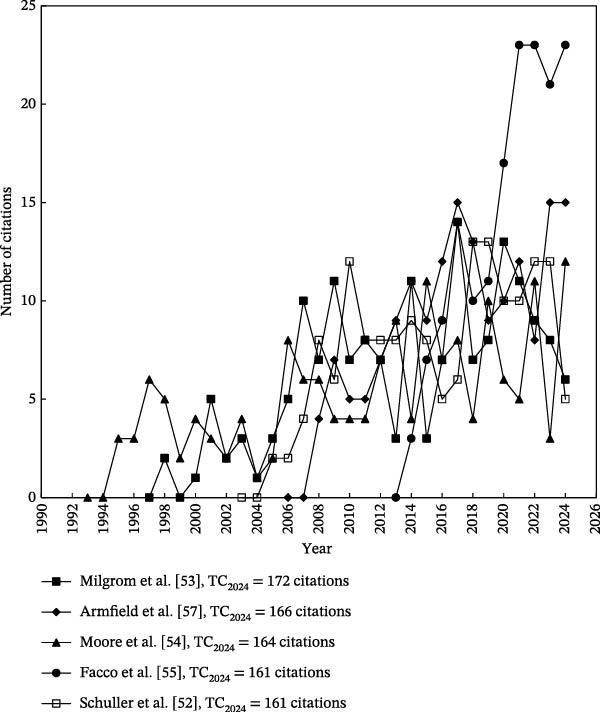
Citation histories of the top 6–10 most frequently cited articles. The sustained citation activity of these publications reflects the broader depth of influential work within the field, extending beyond the most widely cited cornerstone studies.

The citation trajectories of the 10 most highly cited articles on dental anxiety are illustrated in Figures 4 and 5. It is important to note that highly cited articles do not always maintain high annual citation rates overtime [42]. For instance, the article titled “Are there differences in oral health and oral health behavior between individuals with high and low dental fear” by Schuller et al. [52] accumulated a total of 161 citations by 2024 (TC_2024_), ranking 9^th^ among dental anxiety‐related articles. However, in 2024, it received only five citations (*C*_2024_), placing it at 174th for that year (Figure 5). Similarly, the article by Milgrom et al. [53] also demonstrated low recent‐year impact, with a *C*_2024_ of six citations.

Table 6 lists the 10 most frequently cited articles. The citation indicator TC_2024_ was employed to identify the most highly cited articles overall, while *C*_2024_ was used to assess the articles with the highest citation impact in the most recent year within the field. Notably, only four of the 10 most frequently cited articles also appeared among the top 10 most impactful articles in 2024. These four articles are summarized later:

**Table 6:** Top 10 most frequently cited dental anxiety‐related articles.

Rank (TC_2024_)	Rank (*C*_2024_)	Title	Country	Reference
1 (275)	1 (24)	Ambient odors of orange and lavender reduce anxiety and improve mood in a dental office	Austria, Germany	Lehrner et al. [50]
2 (191)	28 (11)	Age of onset of dental anxiety	Canada	Locker et al. [9]
3 (186)	5 (16)	Prevalence of dental fear and phobia relative to other fear and phobia subtypes	The Netherlands	Oosterink et al. [56]
4 (183)	52 (9)	Prevalence of dental anxiety in an adult‐population in a major urban area in Sweden	Sweden	Hakeberg et al. [51]
5 (179)	41 (10)	Child dental fear: cause‐related factors and clinical effects	Sweden	Klingberg et al. [6]
6 (172)	128 (6)	Four dimensions of fear of dental injections	USA	Milgrom et al. [53]
7 (166)	9 (15)	Dental fear in Australia: who’s afraid of the dentist	Australia	Armfield et al. [57]
8 (164)	19 (12)	Prevalence and characteristics of dental anxiety in Danish adults	China, Denmark	Moore et al. [54]
9 (161)	174 (5)	Are there differences in oral health and oral health behavior between individuals with high and low dental fear	Netherlands, Norway	Schuller et al. [52]
9 (161)	2 (23)	Validation of visual analogue scale for anxiety (VAS‐A) in preanesthesia evaluation	Italy	Facco et al. [55]

*Note:* TC_2024_: total number of citations from WoSCC since publication year to the end of 2024; *C*_2024_: number of citations of an article in 2024 only.

1. Ambient odors of orange and lavender reduce anxiety and improve mood in a dental office [50].

The article published by five authors from Austria and Germany with a TC_2024_ of 275 citations (rank 1^st^) and a *C*_2024_ of 24 citations (rank 1^st^). This study investigated whether ambient odors of orange and lavender could reduce anxiety and improve mood among patients in a dental office setting—a context frequently associated with elevated stress. Building on previous research suggesting that certain essential oils may influence emotional states, the authors designed a controlled, between‐subjects experiment involving 200 dental patients aged 18–77 years. Participants were assigned to one of four groups: exposure to orange odor, lavender odor, uplifting music, or no sensory stimulation (control). While waiting for dental treatment, patients completed validated self‐report measures assessing state anxiety and emotional well‐being, including mood, alertness, and calmness. Statistical analyses revealed that both the orange and lavender odor conditions led to significantly lower levels of state anxiety and higher ratings of mood and calmness compared to the control group. In contrast, music did not significantly differ from the control condition on these measures. No significant group differences were found for alertness, and no interactions with gender were detected. These findings provide empirical support for the sedative and mood‐enhancing properties of essential oils before a dental visit and suggest that incorporating natural ambient odors in dental offices may offer a simple, noninvasive method to reduce anticipatory anxiety and promote emotional well‐being in a nonclinical population before a dental visit.

2. Prevalence of dental fear and phobia relative to other fear and phobia subtypes [56].

The article published by three authors from Netherlands with a TC_2024_ of 186 citations (rank 3^rd^) and a *C*_2024_ of 16 citations (rank 5^th^). This study is among the earliest to examine dental phobia, that is, the distinct medical condition, rather than dental anxiety, within a large and representative population sample. The study assessed the point prevalence of dental fear and dental phobia in comparison to 10 other common fears and subtypes of specific phobia, based on DSM‐IV‐TR diagnostic criteria. A representative sample of 1959 Dutch adults (aged 18–93) completed a structured questionnaire evaluating the presence, severity, and psychological correlates of various fears. Participants who endorsed a specific fear completed a validated Phobia Checklist assessing DSM–based criteria and trauma‐related symptoms, including intrusive reexperiencing. The results showed that 24.3% of participants reported dental fear, making it the fourth most common fear after snakes, heights, and physical injuries. Dental phobia was identified in 3.7% of the sample, making it the most prevalent specific phobia subtype. Dental fear was also rated as the most severe and was most strongly associated with intrusive re‐experiencing (49.4%), suggesting a potential trauma‐related dimension. Women more frequently reported fear of dental treatment, although gender differences were not significant for dental phobia prevalence. The findings underscore that while dental fear, especially the severe form of dental phobia, is not the most widespread fear, it appears to be among the most intense and psychologically burdensome. The authors highlight the need to recognize dental phobia as a serious and distinct mental health condition that may warrant specialized clinical attention

3. Dental fear in Australia: who is afraid of the dentist [57].

The article published by three authors from the U Adelaide in Australia with a TC_2024_ of 166 citations (rank 7^th^) and a *C*_2024_ of 15 citations (rank 9^th^). The purpose of this study was to determine the prevalence of dental fear in Australia and to explore its association with demographic, socioeconomic, oral health, insurance, and dental service utilization factors. Using data from the 2002 National Dental Telephone Interview Survey, researchers conducted structured telephone interviews with a nationally representative sample of 7312 individuals aged 5 years and older. Dental fear was assessed using a validated single‐item question. Results indicated that 16.1% of the Australian population reported high dental fear, with higher rates observed among women, individuals aged 40–64, and those from lower socioeconomic backgrounds. Dentate individuals reported significantly more fear than those who were edentulous, and people with high dental fear tended to have more missing teeth, less frequent dental visits, and longer intervals since their last dental appointment. Interestingly, while dental insurance coverage was only slightly lower among individuals with high fear, time since last dental visit showed a strong association, with fear increasing as time since the last visit lengthened. The study also found that people with lower income, lower education, and unstable employment were more likely to report high dental fear. These authors concluded that the findings underscore the need for tailored public health strategies and clinical interventions aimed at identifying and supporting individuals at greater risk of dental fear and avoidance.

4. Validation of visual analogue scale for anxiety (VAS‐A) in preanesthesia evaluation [55].

The article published by seven authors from the University of Padua in Italy with a TC_2024_ of 161 citations (rank 9^th^) and a *C*_2024_ of 23 citations (rank 2^nd^). This study validated the Visual Analogue Scale for Anxiety (VAS‐A) as a simple and reliable tool for assessing preoperative anxiety in patients undergoing oral surgery. While VAS‐A has been used in several clinical contexts, its accuracy and optimal cutoff threshold had not been clearly established. One hundred patients (median age 49) scheduled for implantology or dental extractions completed the VAS‐A alongside the Corah Dental Anxiety Scale (CDAS), the State–Trait Anxiety Inventory (STAI‐Y1 and Y2), and the Beck depression inventory (BDI). The study found significant correlations between VAS‐A and CDAS, STAI‐Y1, and STAI‐Y2, although data dispersion was noted. VAS‐A did not significantly correlate with BDI, but it identified five out of seven participants with depressive symptoms. ROC curve analysis suggested 46 mm as the optimal threshold for clinically relevant anxiety, showing high sensitivity (83%) and good negative predictive value (87%), though with lower specificity (61%). Interestingly, VAS‐A tended to overestimate anxiety compared to other tools, which the authors suggest may reflect its ability to capture broader anxiety components, including fear of surgical outcomes. The findings support the use of VAS‐A as a fast, practical, and sensitive screening tool for preoperative anxiety, making it particularly valuable in clinical anesthetic settings with limited time for psychological assessment.

The four articles with the most impact during the last year of publishing had only a few annual citations during their first years of publication. Notably, the citation trajectories began to rise significantly around 2010, regardless of their original publication dates. This trend aligns with the broad surge of academic interest in dental anxiety, as evidenced by the growing volume of literature on the topic in recent years. The studies by Oostering et al. [56] and Armfield et al. [57] have been particularly impactful in establishing the epidemiological and psychological underpinnings of dental anxiety and dental phobia. Their work on prevalence, correlations, and psychosocial consequences has sustained long‐term relevance and continues to inform research. In contrast, the two more recent contributions by Lehrner et al. [50] and Facco et al. [55] have introduced innovative methods that reflect emerging trends in the field. Facco et al. [55] described an accessible method for quantifying dental anxiety in clinical research. Lehrner et al. [50] were among the first to empirically demonstrate the potential of aromatherapy as a non‐invasive intervention for reducing dental anxiety. These studies were at the forefront of a notable shift toward intervention‐focused research, and a marked increase in publications from countries such as India and China, where interest in complementary approaches, such as aromatherapy and other comfort‐enhancing modalities, has grown substantially. Taken together, these four papers represent articles on different important topics of dental anxiety and their foundational role in shaping contemporary research directions, methodological approaches, and clinical practices.

### 3.6. Research Foci

Over the past 15 years, Ho’s research group has developed innovative methodologies for analyzing word distributions in article titles, abstracts, author keywords, and *Keywords Plus* to identify research foci and emerging trends [22, 58]. The extracted keywords were subsequently compiled into a word bank, which formed the basis for identifying major thematic areas and tracing their evolution overtime [58]. The 1556 articles included in this study were distributed across three distinct periods, each comprising a comparable number of publications: 511 articles from 1991 to 2011, 530 articles from 2012 to 2019, and 515 articles from 2020 to 2024. Excluding the original search terms, the 21 most frequently used author keywords in dental anxiety research and their distribution across the three subperiods (1991–2011, 2012–2019, and 2020–2024) are presented in Table 7.

**Table 7:** Top 21 most frequency used author keywords during 1991–2024.

Author keywords	TP	91–24 *R* (%) *n* = 1556	91–11 *R* (%) *n* = 511	12–19 *R* (%) *n* = 530	20–24 *R* (%) *n* = 515
Oral health	105	1 (8.3)	2 (5.7)	1 (7.3)	1 (11)
Children	84	2 (6.6)	5 (4.4)	1 (7.3)	2 (7.8)
Pain	75	3 (5.9)	2 (5.7)	3 (5.9)	3 (6.1)
Dental care	56	4 (4.4)	1 (7.3)	7 (3.8)	10 (2.6)
Dental caries	51	5 (4.0)	7 (3.4)	6 (4.2)	5 (4.4)
Epidemiology	43	6 (3.4)	4 (5.2)	5 (4.5)	47 (0.87)
Child	42	7 (3.3)	18 (1.8)	4 (4.7)	8 (3.3)
Pediatric dentistry	38	8 (3.0)	37 (1.0)	8 (3.1)	4 (4.6)
Quality of life	29	9 (2.3)	18 (1.8)	10 (2.3)	10 (2.6)
Adolescents	26	10 (2.1)	12 (2.1)	10 (2.3)	19 (1.7)
Prevalence	25	11 (2.0)	9 (2.9)	17 (1.9)	29 (1.3)
Depression	24	12 (1.9)	37 (1.0)	9 (2.6)	13 (2.0)
Sedation	24	12 (1.9)	28 (1.3)	26 (1.4)	9 (2.8)
Oral health‐related quality of life	23	14 (1.8)	37 (1.0)	10 (2.3)	13 (2.0)
Psychometrics	23	14 (1.8)	6 (4.2)	19 (1.6)	N/A
Virtual reality	23	14 (1.8)	169 (0.26)	75 (0.7)	6 (4.1)
Dental treatment	22	17 (1.7)	28 (1.3)	13 (2.1)	19 (1.7)
Behavioral science	21	18 (1.7)	7 (3.4)	19 (1.6)	248 (0.22)
Caries	20	19 (1.6)	18 (1.8)	49 (0.94)	13 (2.0)
Psychology	20	19 (1.6)	10 (2.6)	26 (1.4)	47 (0.87)
Reliability	20	19 (1.6)	12 (2.1)	13 (2.1)	80 (0.65)

*Note:* TP: number of articles; %: percentage in each period; *R*: rank in each period.

The authors identified five overarching subjects in the research of dental anxiety [1]: etiology and risk factors [2], clinical presentation and consequences of dental anxiety [3], prevalence and assessment tools [4], treatment and preventive interventions [5], and pediatric dentistry.

1. Etiology and risk factors deal with the origins and causes of dental anxiety. The topic investigates how dental anxiety develops through a multifactorial interplay of psychological, environmental, and biological factors. Key contributors include negative past dental experiences, trait anxiety, and other personal factors, and forms of vicarious and observational learning. Identifying these risk factors is critical for understanding why dental anxiety develops and can therefore inform the design of preventive interventions aimed at reducing its incidence.
  Supporting words: Disorders, Experience, Acquisition, Experiences, Onset, Predictors, Origins, Memory, Childhood, Conditioning experiences, Personality, Etiology, Cognitive vulnerability, Determinants, Risk‐factors.
2. Clinical presentation and consequences of dental anxiety cover how dental anxiety manifests itself and its consequences. The clinical presentation is characterized by heightened autonomic arousal, avoidance behaviors, and emotional distress. Many times, it is also accompanied by cognitive patterns such as catastrophizing, attention biases, and negative appraisals. The avoidance behaviors lead to delayed or avoided dental visits, resulting in the progression of oral diseases, increased treatment complexity, and a diminished quality of life. Furthermore, this cycle of avoidance maintains or reinforces the anxiety, creating a self‐perpetuating pattern that poses substantial challenges for both patients and practitioners.
  Supporting words: Avoidance, Health, Impact, Oral health, Consequences, Oral health‐related quality of life, Symptoms, Responses, Cognitions.
3. Prevalence and assessment tools investigate how widespread dental anxiety is in a population, and the development of instruments for accurate identification. Epidemiological studies consistently report high prevalence rates, with variations influenced by age, gender, and cultural context. The development and validation of psychometric instruments aids in this, but the instruments measuring dental anxiety are also used as outcome measurements in intervention studies.
  Supporting words: Prevalence, Scale, Validity, Reliability, Population, Validation, Version, Psychometrics, Subscale, Questionnaire, Survey schedule, Anxiety scale, Fear survey schedule, Psychometric properties, Adaptation, Fear survey, Dental anxiety scale.
4. Treatment and preventive interventions evaluate how dental anxiety can be treated or prevented in different populations. The treatment and prevention of dental anxiety encompass a diverse range of interventions aimed at reducing anxiety levels and improving patient engagement with dental care. These strategies include psychological approaches such as exposure‐based CBT for more severe forms of dental anxiety, including the distinct medical condition dental phobia, dental behavioral support (management), as well as pharmacological options like sedation and anxiolytics. Additionally, nontraditional modalities, such as aromatherapy and virtual reality, have gained increasing attention as adjunctive measures in recent years. Preventive efforts focus on early identification, patient education, and the implementation of anxiety‐reducing practices within dental environments.
  Supporting words: Management, Sedation, Virtual reality, Therapy, Midazolam, Efficacy, Anesthesia, Intervention, Follow‐up, Nitrous‐oxide, Premedication, Prevention, Relaxation, Strategies, Propofol, Acupuncture, Applied relaxation, Audiovisual distraction, Virtual‐reality, Conscious sedation, Randomized controlled‐trial, Applied tension, Aromatherapy, Cognitive therapy, Group‐therapy, Individual desensitization, Cognitive‐behavioral therapy, Controlled‐trial, Dexmedetomidine, Diazepam, Double‐blind, Hypnosis, Interventions, Lavender.
5. Pediatric dentistry focuses on dental anxiety in children and adolescents. Pediatric dentistry is the dental specialty that has shown sustained interest in the study and management of dental anxiety, as this condition frequently originates in childhood and demonstrates its highest prevalence among pediatric populations. Addressing dental anxiety is an integral component of pediatric dental care, and specialists in pediatric dentistry often treat patients unable to receive dental care from the general practitioners. Early dental experiences are critical in shaping long‐term attitudes toward oral health, with negative encounters often serving as precursors to persistent dental anxiety and avoidance behaviors. This intersection has positioned dental anxiety as a key focus of research within the specialty, prompting continued research into age‐appropriate assessment tools, intervention, and preventive strategies tailored to children and adolescents.
  Supporting words: Children, Child, Pediatric dentistry, Adolescents, Behavior management problems, Management problems, Distraction, Pediatric dentistry.

**Figure 6:**
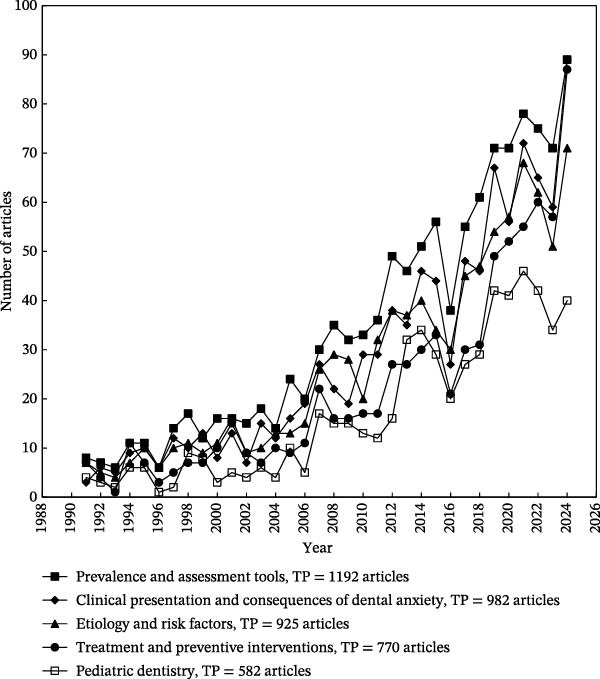
Development trends for five main research topics in dental anxiety research. The thematic shift from epidemiological mapping toward preventive, psychological, and patient‐centered interventions illustrates the maturation of dental anxiety research into a more integrative and clinically relevant domain.

The development trends of the five primary research topics in dental anxiety, as presented in Figure 6, illustrate notable shifts and progressions within the field over the past three decades, highlighting implications for future research and clinical practice.

The first topic, etiology and risk factors, has experienced sustained scholarly attention throughout the periods examined, reflecting the fundamental importance of understanding the origins of dental anxiety. Research consistently underscores the multifactorial nature of dental anxiety, emphasizing psychological variables such as past traumatic dental experiences and cognitive vulnerabilities, including catastrophic thinking and memory biases [9, 59]. Recent literature suggests an increased focus on cognitive and conditioning experiences in childhood, highlighting the critical role of early experiences in shaping long‐term dental anxiety [4]. This underscores the importance of preventive strategies initiated early in life to mitigate future anxiety.

In terms of clinical presentation and consequences of dental anxiety, the research has progressively expanded, reflecting increased recognition of how dental anxiety negatively impacts both oral health and overall quality of life. Avoidance behaviors, commonly associated with dental anxiety, have been shown to affect anxiety and exacerbate oral health deterioration, thus, compounding the complexity and invasiveness of required treatments [60, 61]. Recent findings further suggest that dental anxiety is intertwined with broader psychosocial dimensions, including body image concerns and reduced oral health‐related quality of life, with notable gender‐specific differences observed across populations [62]. Recent evidence also indicates that psychological factors influencing oral‐health perception, such as the role of self‐perceived halitosis across adolescence and adulthood, may intersect with dental anxiety, reinforcing the need to consider emotional, social, and cultural dimensions when evaluating patient distress [63]. Although conscious sedation has been proposed as an effective strategy for managing anxiety during invasive procedures, recent evidence indicates that sedation alone may not adequately address the underlying psychological drivers of dental anxiety [64]. This reinforces the need for complementary behavioral and multidisciplinary approaches, particularly for patients whose anxiety manifests as pronounced avoidance or heightened physiological reactivity.

The third topic, prevalence and assessment tools, remains critically important, with consistent advancements in methodological accuracy and instrument development. Studies show variations in prevalence based on demographic and cultural factors, and although the reliability of psychometric tools is generally high, the construct of dental anxiety that the instruments measure varies significantly [59, 65]. The continual refinement of assessment instruments enhances early detection and accurate measurement of dental anxiety, fundamental for both epidemiological research and intervention effectiveness.

Research related to treatment and preventive interventions has exhibited a marked increase, particularly from 2012 and onward. This likely reflects greater integration of multidisciplinary approaches, combining psychological, pharmacological, and novel adjunctive therapies such as virtual reality and aromatherapy [66, 67]. The COVID‐19 pandemic has further highlighted the complex relationship between clinical protocols and patient anxiety. While newly implemented safety regulations were intended to reassure patients and improve clinical management, recent findings indicate that such measures may paradoxically increase avoidance among highly anxious or phobic individuals, underscoring the need for tailored communication strategies and renewed research efforts [68]. The rise of CBT strategies and the use of different sedation methods highlight a shift towards individualized treatment plans to enhance patient comfort and treatment outcomes.

Collectively, these bibliometric trends point toward a gradual, but clinically meaningful shift toward more integrated and biopsychosocial models of care for dental anxiety. Despite increasing awareness of dental anxiety and its consequences, psychological interventions remain underutilized in many dental settings, creating a persistent gap between patient needs and routine clinical practice. The growing research focus on multidisciplinary approaches, encompassing behavioral therapies, virtual reality‐based distraction, and complementary interventions, suggests an emerging effort to bridge this gap. From a translational perspective, these trends reflect a move beyond symptom management toward addressing the underlying psychological mechanisms of dental anxiety, with important implications for everyday dental care.

Finally, the field of pediatric dentistry demonstrates significant and consistent growth in interest, reflecting increased recognition of childhood as a critical period for addressing dental anxiety. Pediatric dentistry has prioritized behavioral management strategies and preventive interventions aimed at reducing anxiety and improving children’s dental experiences, as negative early encounters can predispose individuals to chronic dental anxiety [6]. Future research will likely continue emphasizing tailored interventions that accommodate developmental stages and specific clinical groups such as those with neurodevelopmental disorders, thereby promoting long‐term positive attitudes toward dental care. These thematic developments reflect a broader clinical transition toward multidisciplinary and nonpharmacological management of dental anxiety. Emerging approaches such as CBT, exposure‐based techniques, virtual‐reality distraction, and aromatherapy illustrate the growing integration of psychological and sensory‐modulating strategies into dental care [3, 50, 69]. When incorporated into routine clinical workflows, these methods can help clinicians reduce anticipatory anxiety, support patient cooperation, and prevent treatment avoidance, ultimately improving both short‐ and long‐term oral health outcomes.

Taken together, the historical trends observed in Figure 6 emphasize a clear progression towards a more integrated, patient‐centered, and multidisciplinary approach to dental anxiety research and clinical practice. Continued attention to these five topics, particularly preventive and pediatric‐focused interventions, will be essential for further advancement in managing dental anxiety effectively. These findings align with broader bibliometric observations reported in independent evaluations of scientific fields, where thematic clustering and citation aging curves reflect the structural maturation of a research domain [5, 14].

## 4. Conclusion

This comprehensive bibliometric study provided insights into the dynamic progression of dental anxiety research from 1991 to 2024. The analysis underscored an increased interest in the topic in recent years and significant thematic expansions and methodological complexity, particularly in the development of assessment tools and intervention strategies. A notable increase in multidisciplinary treatments, including cognitive–behavioral approaches, pharmacological aids, and innovative adjunctive therapies such as virtual reality and aromatherapy, characterized recent publications. Furthermore, pediatric dentistry emerged prominently as a critical domain, emphasizing the necessity of early and tailored interventions to mitigate long‐term dental anxiety. The evolving research underscores a shift towards multidisciplinary, patient‐centered practices, enhancing oral health outcomes and patient comfort. Future investigations should continue prioritizing interdisciplinary approaches, particularly those integrating psychological interventions and emerging technologies, to further improve patient management and preventive strategies. Continued focus on prevention and early evidence‐based treatments for affected individuals in pediatric populations remains essential for addressing dental anxiety at its developmental stages, ultimately promoting lifelong positive attitudes toward dental care, thereby greatly improving oral health.

## Author Contributions

Yuh‐Shan Ho, Robert Vautard, Robert Schibbye, and Nikolaos Christidis contributed to the conceptualization and methodology. Yuh‐Shan Ho and Robert Vautard contributed to data collection, data curation, and formal data analysis. Yuh‐Shan Ho, Robert Schibbye, and Nikolaos Christidis contributed to interpretation. Robert Schibbye and Nikolaos Christidis wrote the original draft, while Yuh‐Shan Ho and Robert Vautard reviewed the manuscript.

## Acknowledgments

No artificial intelligence has been used in any part of the analysis or writing.

## Funding

No funding was received for this manuscript.

## Disclosure

A preprint of this paper has previously been published (Ho et al. [32]; https://www.medrxiv.org/content/10.1101/2025.06.18.25329900v1). All authors have read and revised the manuscript prior to submission.

## Conflicts of Interest

The authors declare no conflicts of interest.

## Data Availability

The data that support the findings of this study are freely available from the Web of Science and the corresponding author upon reasonable request.
